# Comparison of sleep quality based on direction of shift rotation in electronics workers

**DOI:** 10.1186/s40557-016-0122-3

**Published:** 2016-09-05

**Authors:** Youil Shon, Seungho Ryu, Byung-Seong Suh, Soo-Geun Kim, Won-Sool Kim, Hee-Seung Son, Hee-Yun Kim, Han-Seur Jeong

**Affiliations:** 1 Department of Occupational and Environmental Medicine, Kangbuk Samsung Hospital, Sungkyunkwan University School of Medicine, Seoul, Korea; 2 Center for Cohort Studies, Total Healthcare Center, Kangbuk Samsung Hospital, Sungkyunkwan University School of Medicine, Seoul, Korea; 3 Department of Clinical Research Design & Evaluation, SAIHST, Sungkyunkwan University, Seoul, Korea

**Keywords:** Shiftwork, Shift rotation, Sleep quality, Electronics workers

## Abstract

**Background:**

Previous studies have reported the effects of direction of shift rotation on sleep, however, the findings are inconsistent. In this study, we investigated sleep quality related to direction of shift rotation using large-scale data from shiftwork-specific health examinations of electronics workers.

**Methods:**

This study included 4750 electronics workers working in a rotating 3-shift system who completed a medical examination for shift workers survey from January 1 to December 31, 2014, at a general hospital. The subjects were categorized into one of two groups according to direction of shift rotation. We compared sleep quality index between the subjects who worked in forward rotation and backward rotation systems.

**Results:**

Backward rotation was positively associated with prevalence of poor sleep quality. In the multivariable-adjusted model, when comparing backward rotation to forward rotation, the odds ratio (OR) with 95 % confidence interval (95 % CI) for poor sleep quality was 1.95 (1.58–2.41). After stratifying by gender, the ORs (95 % CIs) for poor sleep quality in male and female was 1.92 (1.47–2.49) and 2.13 (1.47–3.08), respectively. In subgroup analyses, backward rotation was significantly associated with poor sleep quality in workers ≥30 years of age compared with workers <30 years of age (adjusted OR 2.60 vs. 1.89, respectively; *P* for interaction <0.001).

**Conclusions:**

Our study supports that a backward rotation system is associated with poor sleep quality. Forward rotation systems should be considered to reduce sleep problems.

## Background

Many epidemiologic studies have reported that shift work increases the risk of accidents and can affect the cardiovascular, gastrointestinal, endocrine systems, and mental health [1–3]. Disruption of circadian rhythm is a key factor mediating the adverse health and safety outcomes observed among shift workers [3, 4]. To decrease the disruption of circadian rhythm, the application of healthier shift systems has been suggested [5, 6]. Forward rotation is a strategy for decreasing disruption of circadian rhythm [5–7]. This type of shift system first moves from a morning shift to an evening shift and then to a night shift (also called phase delay or clockwise rotation). Backward rotation (night to evening to morning) is called counterclockwise rotation or phase advance. Compared with forward rotation shift systems, backward rotation systems have been associated with poorer health outcomes, including more unfavorable shift of the autonomic balance [8], adverse changes in serum triglyceride, glucose, and systolic blood pressure [9], fatigue [7, 10], and increased need for recovery from sickness or injury [10].

Several studies have reported the effects of direction of shift rotation on sleep, however, the findings are inconsistent [9–13]. Several studies have reported that backward rotation systems were associated with poorer sleep outcomes such as sleep quality [9, 10] and sleep-wake cycle [11], while others reported no or limited relationship [12, 14]. In addition, prior studies used small sample sizes [9–13], or focused on males [9, 10], and did not consider differences among job types, which may have biased the results [10, 12].

Therefore, we investigated sleep quality related to direction of shift rotation, using large-scale data from shiftwork-specific health examinations of electronics workers. To evaluate the effect of direction of shift rotation on sleep quality, we compared sleep quality indexes among subjects who worked in forward rotation and backward rotation systems. In addition, we performed subgroup analyses to identify the factors associated with the effect of shift work direction on sleep quality.

## Methods

### Subjects

The participants were selected from shift workers who worked in a rotating 3-shift system at 3 electronics manufacturing plants (day work from 6:00 to 14:00, evening work from 14:00 to 22:00, night work from 22:00 to 06:00). Shift workers have original schedule of shift rotation based on the schedule for their 4-team 3-shift. However, direction of shift rotation can be different because schedule of shift rotation can be modified by supervisor. A total of 4891 shift workers who received a survey questionnaire regarding the specific medical examination of shift workers at a general hospital from January 1 to December 31, 2014, were eligible for our study. Of those, 85 subjects who did not complete the questionnaire were excluded. Among the remaining 4806 subjects, exclusion criteria included height or weight not measured (*n* = 13) and subjects diagnosed with or treated for specific diseases such as stroke (*n* = 5), cardiovascular disease (*n* = 12), or diabetes (*n* = 30) [15]. Because some individuals met more than one exclusion criterion, the total number of patients eligible for this study was 4750. The subjects were categorized into one of two groups according to direction of shift rotation. This study was approved by the Institutional Review Board of Kangbuk Samsung Hospital, and the requirement for informed consent was waived because we used de-identified retrospective data routinely collected during the health screening process.

### Measurements

A standardized self-reported questionnaire was administered to obtain information on shift work including duration of work experience, type, regularity, weekly working hours, and direction of shift rotation, sex, age, smoking status, alcohol consumption, physical activity, and sleep quality.

Smoking status was categorized into the following three groups. Subjects who had smoked <100 cigarettes in their lifetime were grouped as non-smokers. Subjects who smoked at least 100 cigarettes in their lifetime were further classified into ex-smokers (stopped smoking) or current smokers (currently smoking). Subjects were classified as non-drinkers, regular drinkers (male, ≤2 drinks/day; female, ≤1 drink/day), or heavy drinkers (male, >2 drinks/day; female, >1 drink/day) [16]. In addition, to obtain information on physical activity, subjects were asked to report the frequency of physical activity in one week based on two categories by intensity: vigorous (at least 20 min a day) or moderate (at least 30 min a day). Physical activity was categorized as sufficient exercise (vigorous activity time × 2 + moderate activity time ≥150 min in one week) or insufficient exercise (vigorous activity time × 2 + moderate activity time <150 min in one week) [17]. Lastly, the duration of shift work was categorized as <5 years, 5–9 years, or ≥10 years. Height and weight were measured by trained nurses. Body mass index (BMI) was calculated as weight in kilograms divided by height in meters squared.

We obtained the sleep quality index from the Pittsburg Sleep Quality Index (PSQI), a self-reported questionnaire that measures retrospective sleep quality and disturbances over a one-month period. PSQI consists of 19 questions that assess a wide variety of factors related to sleep quality, including estimates of sleep duration and latency as well as frequency and severity of specific sleep-related problems. The 19 items are grouped into seven component scores, each weighted equally on a 0–3 scale. The seven component scores are then summed to yield a global PSQI score ranging from 0 to 21; a total score of 5 or less indicates “good” sleep, and scores greater than 5 indicates “poor” sleep [18].

### Statistical analysis

Characteristics of the study participants were evaluated according to the groups of direction of shift rotation. In addition, general characteristics of the male and female workers were evaluated according to the groups of direction of shift rotation. To assess differences across the groups, a Chi-square tests were used for categorical data and Mann-Whitney tests for continuous data.

To evaluate the association between direction of shift rotation and sleep quality, we used binomial logistic regression models to estimate odds ratios (ORs) with 95 % confidence intervals (95 % CIs) for poor sleep quality. We initially adjusted for age and sex and further adjusted for BMI, duration of shift work, weekly working hours, smoking status, alcohol consumption, and physical activity. We further evaluated the association between direction of shift rotation and sleep quality after stratification by gender.

We also performed stratified analyses in pre-specified subgroups defined by age (<30 years of age vs. ≥30 years of age), BMI (18.5–24.9 kg/m^2^ vs. <18.5 kg/m^2^ vs. ≥25 kg/m^2^) [19], duration of shift work (<5 years vs. 5–9 years vs. ≥10 years), weekly working hours (<41 h vs. 41–51 h vs. ≥52 h), smoking status (non-smoker vs. ex-smoker vs. current smoker), alcohol consumption (non-drinker vs. regular drinker vs. heavy drinker), and physical activity (sufficient exercise vs. insufficient exercise); interactions between subgroups were tested using likelihood ratio tests comparing models with and without multiplicative interaction terms. Statistical analysis was performed using SPSS Statistics Version 19 (Chicago, IL, USA).

## Results

The baseline characteristics of study participants related to direction of shift rotation are outlined in Table 1 (Table 1). This study included 1749 males (36.8 %) and 3001 females (63.2 %); the mean ± standard deviation (SD) of age, BMI, sleep duration, and PSQI score of the 4750 participants were 27.5 ± 4.4 years, 23.0 ± 4.0 kg/m^2^, 7.0 ± 1.6 h, and 4.4 ± 2.7 points, respectively.

**Table 1:** General characteristics of the study subjects

	Total	Forward rotation	Backward rotation	*P* value^*^
Number of participants	4750	4238 (89.2)	512 (10.8)	
Age, years	27.5 ± 4.4	27.5 ± 4.5	27.9 ± 3.6	0.001
Sex				<0.001
Male	1749	1359 (77.7)	390 (22.3)	
Female	3001	2879 (95.9)	122 (4.1)	
BMI, kg/m^2^	23.0 ± 4.0	22.9 ± 4.1	23.6 ± 3.5	<0.001
Duration of shift work				<0.001
<5 years	1870	1517 (81.1)	353 (18.9)	
5–9 years	1738	1635 (94.1)	103 (5.9)	
≥10 years	1142	1086 (95.1)	56 (4.9)	
Weekly working hours				<0.001
<41 h	1013	933 (92.1)	80 (7.9)	
41–51 h	3115	2835 (91.0)	280 (9.0)	
52–59 h	516	398 (77.1)	118 (22.9)	
≥60 h	106	72 (67.9)	34 (10.8)	
Smoking status				<0.001
Non-smoker	3638	3308 (90.9)	330 (9.1)	
Ex-smoker	1081	903 (83.5)	178 (16.5)	
Current smoker	31	27 (87.1)	4 (12.9)	
Alcohol consumption				<0.001
Non-drinker	1896	1749 (92.2)	147 (7.8)	
Regular drinker	2450	2120 (86.5)	330 (13.5)	
Heavy drinker	404	369 (91.3)	35 (8.7)	
Physical activity				<0.001
Sufficient exercise	1544	1339 (86.7)	205 (13.3)	
Insufficient exercise	3206	2899 (90.4)	307 (9.6)	
Sleep duration, hours	7.0 ± 1.6	7.0 ± 1.6	6.9 ± 1.4	0.011
PSQI score, points	4.4 ± 2.7	4.3 ± 2.7	4.9 ± 2.6	<0.001

Values are number (%) or mean ± Standard Deviation (SD), unless otherwise indicated

*P* value^*^ = Chi-square test or Mann-Whitney test

Of the 4750 subjects, 4238 (89.2 %) were in forward rotation and 512 (10.8 %) in backward rotation. Among the subjects, there were more male workers in backward rotation (76.2 %) than in forward rotation (32.1 %). The mean age, BMI, and PSQI score were higher in backward rotation, whereas mean sleep duration was higher in forward rotation. Statistically significant differences existed among duration of shift work, weekly working hours, smoking status, alcohol consumption, and physical activity.

The mean ± SD of age, BMI, sleep duration, and PSQI score of the 1749 male workers were 28.4 ± 4.5 years, 24.5 ± 3.4 kg/m^2^, 6.8 ± 1.4 h, and 4.1 ± 2.5 points, respectively. Of the 1749 male workers, 1359 (77.7 %) were in forward rotation and 390 (22.3 %) in backward rotation. The mean PSQI score was higher in backward rotation. Statistically significant differences existed among duration of shift work, and weekly working hours (Table 2).

**Table 2:** General characteristics of the male workers

	Total	Forward rotation	Backward rotation	*P* value^*^
Number of participants	1749	1359 (77.7)	390 (22.3)	
Age, years	28.4 ± 4.5	28.4 ± 4.7	28.2 ± 3.4	0.685
BMI, kg/m^2^	24.5 ± 3.4	24.5 ± 3.5	24.2 ± 3.3	0.124
Duration of shift work				<0.001
<5 years	1134	823 (72.6)	311 (27.4)	
5–9 years	414	358 (86.5)	56 (13.5)	
≥10 years	201	178 (88.6)	23 (11.4)	
Weekly working hours				<0.001
<41 h	416	368 (88.5)	48 (11.5)	
41–51 h	947	742 (78.4)	205 (21.6)	
52–59 h	311	206 (66.2)	105 (33.8)	
≥60 h	75	43 (57.3)	32 (42.7)	
Smoking status				0.379
Non-smoker	935	716 (76.6)	219 (23.4)	
Ex-smoker	788	621 (78.8)	167 (21.2)	
Current smoker	26	22 (84.6)	4 (15.4)	
Alcohol consumption				0.534
Non-drinker	384	292 (76.0)	92 (24.0)	
Regular drinker	1251	975 (77.9)	276 (22.1)	
Heavy drinker	114	92 (80.7)	22 (19.3)	
Physical activity				0.454
Sufficient exercise	802	630 (78.6)	172 (21.4)	
Insufficient exercise	947	729 (77.0)	218 (23.0)	
Sleep duration, hours	6.8 ± 1.4	6.8 ± 1.4	6.9 ± 1.4	0.158
PSQI score, points	4.1 ± 2.5	3.9 ± 2.5	4.7 ± 2.5	<0.001

Values are number (%) or mean ± Standard Deviation (SD), unless otherwise indicated

*P* value^*^ = Chi-square test or Mann-Whitney test

The mean ± SD of age, BMI, sleep duration, and PSQI score of the 3001 female workers were 27.0 ± 4.3 years, 22.1 ± 4.1 kg/m^2^, 7.1 ± 1.6 h, and 4.6 ± 2.8 points, respectively. Of the 3001 female workers, 2879 (95.9 %) were in forward rotation and 122 (4.1 %) in backward rotation. The mean PSQI score was higher in backward rotation, whereas mean sleep duration was higher in forward rotation. Statistically significant differences existed among duration of shift work (Table 3).

**Table 3:** General characteristics of the female workers

	Total	Forward rotation	Backward rotation	*P* value^*^
Number of participants	3001	2879 (95.9)	122 (4.1)	
Age, years	27.0 ± 4.3	27.0 ± 4.3	27.0 ± 4.1	0.870
BMI, kg/m^2^	22.1 ± 4.1	22.1 ± 4.1	21.7 ± 3.4	0.357
Duration of shift work				0.034
<5 years	736	694 (94.3)	42 (5.7)	
5–9 years	1324	1277 (96.5)	47 (3.5)	
≥10 years	941	908 (96.5)	33 (3.5)	
Weekly working hours				0.051
<41 h	597	565 (94.6)	32 (5.4)	
41–51 h	2168	2093 (96.5)	75 (3.5)	
52–59 h	205	192 (93.7)	13 (6.3)	
≥60 h	31	29 (93.5)	2 (6.5)	
Smoking status				0.900
Non-smoker	2703	2592 (95.9)	111 (4.1)	
Ex-smoker	293	282 (96.2)	11 (3.8)	
Current smoker	5	5 (100.0)	0 (0.0)	
Alcohol consumption				0.497
Non-drinker	1512	1457 (96.4)	55 (3.6)	
Regular drinker	1199	1145 (95.5)	54 (4.5)	
Heavy drinker	290	277 (95.5)	13 (4.5)	
Physical activity				0.592
Sufficient exercise	742	709 (95.6)	33 (4.4)	
Insufficient exercise	2259	2170 (96.1)	89 (3.9)	
Sleep duration, hours	7.1 ± 1.6	7.2 ± 1.6	6.8 ± 1.5	0.037
PSQI score, points	4.6 ± 2.8	4.5 ± 2.7	5.6 ± 2.8	<0.001

Values are number (%) or mean ± Standard Deviation (SD), unless otherwise indicated

*P* value^*^ = Chi-square test or Mann-Whitney test

Table 4 shows the association between direction of shift rotation and sleep quality. Of 4750 subjects included in this study, 1382 had poor sleep quality. Backward rotation was positively associated with prevalence of poor sleep quality. In the age- and sex-adjusted model, when comparing backward rotation to forward rotation, the OR (95 % CI) for poor sleep quality was 2.01 (1.64–2.47). In a multivariate model adjusting for potential confounders, backward rotation was still significantly associated with increasing risk of poor sleep quality. When comparing backward rotation to forward rotation for poor sleep quality, the corresponding OR (95 % CI) was 1.95 (1.58–2.41).

**Table 4:** Odds ratio and 95 % CI for poor sleep quality according to direction of shift rotation

Direction of shift rotation	Number of participants	Cases of poor sleep quality	Age- and sex- adjusted OR (95 % CI)	Multivariate- adjusted OR (95 % CI)
Total				
Forward rotation	4238	1189	Reference	Reference
Backward rotation	512	193	2.01 (1.64–2.47)	1.95 (1.58–2.41)
Male				
Forward rotation	1359	287	Reference	Reference
Backward rotation	390	133	2.02 (1.57–2.59)	1.92 (1.47–2.49)
Female				
Forward rotation	2879	902	Reference	Reference
Backward rotation	122	60	2.12 (1.48–3.05)	2.13 (1.47–3.08)

Multivariable model adjusted for age, sex, BMI, duration of shift work, weekly working hours, smoking status, alcohol consumption, and physical activity

*OR* odds ratio, *95 % CI* 95 % confidence interval, *BMI* body mass index

After stratifying by gender, the age- adjusted ORs (95 % CIs) for poor sleep quality in male and female was 2.02 (1.57–2.59) and 2.12 (1.48–3.05), respectively. In a multivariate model adjusting for potential confounders, the ORs (95 % CIs) for poor sleep quality in male and female was 1.92 (1.47–2.49) and 2.13 (1.47–3.08), respectively.

Next, the association between direction of shift rotation and sleep quality was examined in subgroups of study participants (Table 5). Backward rotation was significantly associated with poor sleep quality in workers ≥30 years of age compared with workers <30 years of age (adjusted OR 2.60 vs. 1.89; *P* interaction <0.001). There were no significant interactions based on BMI (18.5–24.9 kg/m^2^ vs. <18.5 kg/m^2^ vs. ≥25 kg/m^2^) [15], duration of shift work (<5 years vs. 5–9 years vs. ≥10 years), weekly working hours (<41 h vs. 41–51 h vs. ≥52 h), smoking status (non-smoker vs. ex-smoker vs. current smoker), alcohol consumption (non-drinker vs. regular drinker vs. heavy drinker), and physical activity (sufficient exercise vs. insufficient exercise).

**Table 5:** Odds ratio and 95 % CI for poor sleep quality based on direction of shift rotation in subgroups

Subgroup	Forward rotation	Backward rotation	*P* value for interaction
Age (years)			<0.001
<30	Reference	1.89 (1.46–2.45)	
≥30	Reference	2.60 (1.77–3.83)	
BMI (kg/m^2^)			0.423
18.5–24.9	Reference	1.84 (1.42–2.40)	
<18.5	Reference	2.56 (1.10–5.96)	
≥25	Reference	2.19 (1.48–3.24)	
Duration of shift work			0.197
<5 years	Reference	1.78 (1.34–2.36)	
5–9 years	Reference	2.48 (1.63–3.77)	
≥10 years	Reference	2.07 (1.19–3.60)	
Weekly working hours			0.417
<41 h	Reference	1.73 (1.03–2.88)	
41–51 h	Reference	1.96 (1.48–2.59)	
≥52 h	Reference	1.82 (1.19–2.79)	
Smoking status			0.566
Non-smoker	Reference	1.95 (1.51–2.53)	
Ex-smoker	Reference	1.99 (1.38–2.88)	
Current smoker	Reference	5.36 (0.43–67.26)	
Alcohol consumption			0.508
Non-drinker	Reference	1.76 (1.20–2.58)	
Regular drinker	Reference	1.91 (1.46–2.49)	
Heavy drinker	Reference	3.32 (1.55–7.13)	
Physical activity			0.427
Sufficient exercise	Reference	1.97 (1.40–2.78)	
Insufficient exercise	Reference	1.94 (1.49–2.53)	

Models were adjusted for age, sex, BMI, duration of shift work, weekly working hours, smoking status, alcohol consumption, and physical activity

*OR* odds ratio, *95 % CI* 95 % confidence interval, *BMI*, body mass index

## Discussion

In this study, we investigated sleep quality related to direction of shift rotation using large-scale data from shift work-specific health examinations of electronic workers. Our results showed that backward rotation system was associated with poor sleep quality. Additionally, backward rotation system was significantly associated with poor sleep quality in workers ≥30 years of age compared with workers <30 years of age.

Previous studies have found an association between direction of shift rotation and sleep quality, however, the results are inconsistent. Orth-Gomer assessed sleep quality and coronary risk factors in a short-term intervention trial that included 45 volunteer policemen [9] and reported that sleep was longer and better with forward rotation. Van Amelsvoort et al. reported that a forward rotation schedule was prospectively related to less work-family conflict and better sleep quality over 32 months of follow-up [10]. Our finding is consistent with these previous study results. However, several other studies indicated that direction of shift rotation did not play a significant role in sleep problems [12, 13]. Cruz et al. assessed subjective ratings of sleep quality, sleepiness, and objective source of sleep/wake data using activity sensors in 28 participants [12]. They reported no effect of rotation condition for any of the sleep measures. Tucker et al. found no effect of direction of rotation on any of the chronic outcome measures despite the association between direction of rotation and sleep duration [13].

The exact mechanisms of increasing risk of poor sleep quality by backward rotation system have yet to be elucidated. Previous studies have found that the circadian rhythms, generated by the suprachiasmatic nucleus (SCN) of the anterior hypothalamus in mammals [20, 21], can be synchronized to external time signals but also can persist in the absence of such signals [22, 23]. In normal conditions, the SCN generates circadian rhythms by receiving light inputs from the retina during the day and from melatonin during the night [20, 21], and SCN neuronal activity drives the circadian variation of the sleep/wake cycle, hormonal secretion, thermoregulation, and other physiologic events [20, 24]. Even in the absence of external signals, SCN neurons have a near-24-h rhythm of electrical activity [21, 25]. This circadian activity reflects the rhythmic pattern of expression of core genes, called clock genes, that are regulated by autoregulatory feedback loops [21, 25, 26]. If the circadian rhythms were driven by external signals, they would persist for a period of exactly 24 h. However, without external signals, the circadian rhythm period is slightly longer than 24 h [27]. Most humans already have a natural tendency to drift slightly later each day; therefore, the human circadian rhythm is more difficult to phase-advance than to phase-delay [28, 29]. It takes less time to reset the circadian rhythm following westward (requiring a phase delay) than eastward (requiring a phase advance) flight [30, 31]. Similarly, adaptation is more rapid after forward rotation (requiring a phase delay) than after backward rotation (requiring a phase advance) [28, 29].

Furthermore, previous studies have found that young age was associated with shift work tolerance, measured as subjective sleepiness, performance tests, recovery after work, and sleep time [32–34]. Some studies have indicated that the critical age for reduced tolerance to shift work is between 40 and 50 years [35, 36]. Results from another study showed that both sleep duration and sleep quality among shift workers decreased with increasing age up to approximately 45 years [37]. These findings might be explained by age-related disruptions of circadian rhythms characterized by changes in both behavior and physiology [38]. Age is associated with decreased electrical, hormonal, and gene-expression activity of SCN cells, which are thought to globally disrupt the body’s circadian activity [38–40]. In elderly humans, rhythm disturbances include fragmented sleep–wake patterns, weak coupling with environmental rhythms, reduced amplitude of daily body temperature rhythms, alterations in the daily rhythm of hormone secretion, high levels of nighttime activity, and reduced daytime cognitive performance [38, 41–43]. These disadvantages may partly explain why the backward rotation system was significantly associated with poor sleep quality in older age.

In our study, we showed that forward rotation systems should be considered to reduce sleep problems. Reportedly, sleep problems have a direct association with accidents or errors at work [44]. Furthermore, in shift workers, sleep problems are represented mainly by the disruption of the circadian rhythm, which adversely affects health [3, 4]. Forward rotation systems are thought to reduce not only sleep problems, but also adverse health effects by decreasing disruption of the circadian rhythm.

Our study had several limitations. First, speed and interval of shift rotation were not identified because the data used in the present study were collected from shift work-specific health examinations. Several studies have suggested that fast-rotating shift systems (change of working hours every 2–3 days) are preferable to slow-rotating shift systems [5]. In addition, several studies have suggested that a shorter shift rotation interval leads to worse sleep quality [6]. To decrease variation in shift rotation, we selected subjects who worked in the electronics industry. Second, we did not take into account several confounding factors such as work condition including type of work and resting time during work [14, 45], socioeconomic status [46], marital status [47], time for chores [48], which may have influenced the association between direction of shift rotation and sleep quality. Third, there is a possibility that current-smoker were underestimated because we did not apply time frame to classify for ex-smoker. Fourth, this was a cross-sectional study, therefore, the temporal relationship could not be determined and the causal relationship between direction of shift rotation and sleep quality could not be investigated. Finally, the subjects included in this study were younger-aged Korean males and females who regularly attended work-related health check-up programs. As a consequence, our findings may not be representative of the general Korean population or of other populations with different demographics.

Prior studies used a small sample size [9–13], focused on males [9, 10], and did not consider that differences between job types may have biased the results [10, 12]. In our study, the large-scale data of electronic workers allowed a greater statistical power and showed the association between direction of shift rotation and sleep quality in the electronics industry. Furthermore, previous studies that suggested an association between direction of rotation and sleep quality, assessed sleep quality using non-standardized methods. In this study, we used the PSQI, a reliable, valid, and standardized measure of sleep quality and a screening tool for sleep dysfunction in non-clinical and clinical samples [18, 49].

## Conclusions

Despite several limitations, our study showed that a backward rotation system was associated with poor sleep quality. Furthermore, this association was not interacted by factors such as BMI, duration of shift work, weekly working hours, smoking status, alcohol consumption, and physical activity except the age, which suggests that these factors may not affect the effect of shift work direction on sleep quality. Sleep problems are represented mainly by the disruption of circadian rhythm in shift workers, which adversely affects health. Forward rotation systems reduce sleep problems by decreasing the disruption of the circadian rhythm and should be considered to improve shift workers’ health. Further controlled prospective studies evaluating the effects on shift workers’ physical and mental health including sleep problems are needed without changing other important parameters of the shift system.

## Abbreviations

95 % CI: 95 % confidence interval
BMI: Body mass index
OR: Odds ratio
PSQI: Pittsburg Sleep Quality Index
SCN: Suprachiasmatic nucleus
SD: Standard deviation
